# Fate of tenogenic differentiation potential of human bone marrow stromal cells by uniaxial stretching affected by stretch-activated calcium channel agonist gadolinium

**DOI:** 10.1371/journal.pone.0178117

**Published:** 2017-06-27

**Authors:** Hui Yin Nam, Hanumantha Rao Balaji Raghavendran, Belinda Pingguan-Murphy, Azlina A. Abbas, Azhar M. Merican, Tunku Kamarul

**Affiliations:** 1 Tissue Engineering Group, Department of Orthopaedic Surgery (NOCERAL), Faculty of Medicine, University of Malaya, Kuala Lumpur, Malaysia; 2 Department of Biomedical Engineering, Faculty of Engineering, University of Malaya, Kuala Lumpur, Malaysia; The University of Adelaide, AUSTRALIA

## Abstract

The role for mechanical stimulation in the control of cell fate has been previously proposed, suggesting that there may be a role of mechanical conditioning in directing mesenchymal stromal cells (MSCs) towards specific lineage for tissue engineering applications. Although previous studies have reported that calcium signalling is involved in regulating many cellular processes in many cell types, its role in managing cellular responses to tensile loading (mechanotransduction) of MSCs has not been fully elucidated. In order to establish this, we disrupted calcium signalling by blocking stretch-activated calcium channel (SACC) in human MSCs (hMSCs) *in vitro*. Passaged-2 hMSCs were exposed to cyclic tensile loading (1 Hz + 8% for 6, 24, 48, and 72 hours) in the presence of the SACC blocker, gadolinium. Analyses include image observations of immunochemistry and immunofluorescence staining from extracellular matrix (ECM) production, and measuring related tenogenic and apoptosis gene marker expression. Uniaxial tensile loading increased the expression of tenogenic markers and ECM production. However, exposure to strain in the presence of 20 μM gadolinium reduced the induction of almost all tenogenic markers and ECM staining, suggesting that SACC acts as a mechanosensor in strain-induced hMSC tenogenic differentiation process. Although cell death was observed in prolonged stretching, it did not appear to be apoptosis mediated. In conclusion, the knowledge gained in this study by elucidating the role of calcium in MSC mechanotransduction processes, and that in prolonged stretching results in non-apoptosis mediated cell death may be potential useful for regenerative medicine applications.

## Introduction

The role for mechanical stimulation in the control of cell behaviour has been proposed to include cell proliferation, differentiation and even cell death. It is further suggested that mechanical conditioning of mesenchymal stromal cells (MSCs) should be an area of interest as the manipulation of mechanical signalling cues, which enables MSC behaviour to be directed toward a specific cell lineage, can be potentially useful for tissue engineering applications. Mechanical loading is beneficial to the musculoskeletal system [1], particularly in tendons which it has been shown to produce superior repair outcomes when repaired tendons are subjected to stretching exercises [2–3]. When stretched, tendon cells, or commonly known as tenocytes, receives mechanical stimulation and translates them into biological cues which can be observed as phenotypic expressions [4]. This process is known as mechanotransduction. However, limited in their ability, existing tenocytes may not be sufficient to provide sufficient repair process and thus the need for cells to be recruited to the damaged or repaired site becomes paramount [5–6]. Local progenitor cells such as tendon progenitor cells, or sometimes known as tendon stem cells, provides the cell numbers required to make the repair process more effective [7]. Mechanotransduction also plays a role in this process, as it affects the transformation process of tendon stem cells into tenocytes, i.e. cell tenogenic differentiation. Recruited cells receive not only chemical cues to undergo cellular differentiation but also mechanical cues to direct cells towards tenogenic cell differentiation. It is based on this notion that researchers have embarked on the idea of using stem cells from external sources to provide the desired reparative effects.

In recent years, there has been an explosion in the number of research relating to the use of stem cells and other strategies to exploit this knowledge, i.e. with the aim to improve tendon reparative processes. One strategy, known as tissue engineering, has been introduced to manage tendon injuries, which includes the use of autologous or allogenic tenocytes, and/or autologous or allogenic adult stem cells with or without the use of delivery vehicles or biomaterials into damaged sites [8]. However, there have been several limitations being reported from using this technique, of which it has been shown that because of the loss of cell signalling such as calcium signalling, transplanted cells do not always survive the transplantation procedure, or if it does, it does not produce the desired sustainable effects needed in tendon repair [9–10]. It is thus become apparent that cell signalling is also an important aspect to be considered in pursuing the development of tendon tissue engineering.

In controlling cell signalling, several reports have suggested that calcium (Ca^2+^) as a signalling molecule is of importance, owing to the fact that there are many facets of cell behaviour that involves calcium dependent cell signalling communication and interactions [11–13]. Although previous studies have reported on the role of calcium signalling in many facets of cell physiology and in many cell types, the mechanisms related to the process of mechanotransduction and its relationship in controlling cell fate have not been extensively described. Whilst it may appear less likely that a minor change in the external mechanical cues can be perceived and transmitted at the cellular level, this phenomenon occurs quite frequently [14–16] and is said to involve many cellular pathways and function [17–18].

To explore the possibility of the role for calcium signalling in the management of cellular responses to tensile loading, we investigated the involvement of extracellular-intracellular calcium movement by disrupting the function of stretch-activated calcium channel (SACC) of human MSCs (hMSCs). We then observe the effects on MSC differentiation and death. It is worth noting that whilst calcium signalling may control many facets of cell behaviour, in this study we have limited our investigation to only cell differentiation and cell death; since it is our intention to establish the rate and strain levels that would provide the optimal cell response. This is in line with the study we conducted previously [19].

As mentioned earlier, MSCs sense mechanical force through different mechanisms but are influenced, or even influences calcium movements’ intra- and extra-cellularly during this process [20]. Of several notable calcium unit regulations and mechanisms, stretch-activated ion channels (SACs) has been mentioned as the most likely component to be involved extensively when receiving mechanical stimuli [21]. Mechanical stimulation of cells may create calcium signals by causing the entry of the calcium ion across the plasma membrane or it can be released from intracellular stores [14]. We postulate that changes in cyclic mechanical loading activate stretch-sensitive channels in MSCs and modulate tenogenic differentiation in physiological conditions. We also postulate that in prolonged cyclical conditions it is possible that cell death will occur, but may not be via apoptosis. To study this, we examine the differentiation potential of human bone marrow stromal cells when subjected to uniaxial stretching treated with or without gadolinium and looked at the possibility as to whether apoptosis pathways may be involved from the genes expressed.

## Materials and methods

### Harvesting bone marrow specimens from human

The use of bone marrow stromal cells in this study was based on the approval from the Medical Ethics Committee in University Malaya Medical Centre (reference number: 369.19). Bone marrow specimens were collected using a large aspirator by orthopaedic surgeons from our institution. We obtained written informed consents from ten patients undergoing arthroplasty type procedures (N = 10; mean age = 65 years).

### Isolation and expansion of hMSCs

Human bone marrow MSCs isolation was performed using our standard laboratory protocol, also previously described in other publications [19, 22]. Briefly, diluted bone marrow specimens were slowly layered on top of Ficoll–Paque PREMIUM (density of 1.077 g/mL) (Amersham Biosciences, Uppsala, Sweden). The mononuclear cells were then extracted after undergoing gradient density centrifugation at 2,200 rpm for 25 min. The cell pellet was extracted after a centrifugation at 1,600 rpm for 10 min. The pellet was then resuspended in fetal bovine serum (FBS) (Invitrogen-Gibco, USA). The mixture was then transplanted in culture flask to be cultured in cell culture medium (low-glucose DMEM supplemented with 10% FBS, 1% penicillin-streptomycin and 1% Glutamax-1) (Invitrogen-Gibco, USA). Cell cultures were maintained at 37^°^C (humidified atmosphere) supplemented with 5% CO_2_. Growth medium was replaced every 72 hrs to achieve optimal cell proliferation. This continued until the cell cultures became 80–85% confluent. The cells were serially passaged and expanded until passage-2 before being used for our experiments. The hMSCs surface markers analysis and tri-lineage differentiation was performed following standards established as our standard laboratory protocol, which was also mentioned in our previous publications [19].

### Optimization of gadolinium concentration

A stock solution 10 mM of gadolinium (Sigma, USA) was prepared in distilled water. To optimize the concentration of gadolinium to be used in this study, the blocker at various concentrations (2 μM, 10 μM, 20 μM, 50 μM, 80 μM and 100 μM) was diluted with culture medium immediately before treatment was started on hMSCs. The optimum concentration was chosen and continued for the following experiments.

### Cells seeding and the application of mechanical stretching on cultured cells

A total of 1 x 10^5^ passaged-2 hMSCs per well were seeded onto 10 cm^2^ collagen type I-coated (Sigma, USA) silicone chamber (STREX, Japan). In order to synchronize cells at G_0_ phase of the cell cycle, the concentration of FBS was reduced to 1% after 48 h of the initial culture period. Following synchronization for 24 h, the samples were changed to normal cell culture medium before assembled into uniaxial strain device. The device was programmed to approximate sinusoidal waveforms to 8% strain amplitude at a frequency of 1Hz. This variable was based on our previous findings where we demonstrated that it this setting, cell differentiation was optimal and resulted in enhanced collagen synthesis or tenogenesis gene expression [19]. Cells in the control group were also cultured on silicone chamber and maintained in the same incubator, but did not undergo mechanical stimulation. The cells were harvested after 6, 24, 48, and 72 h of cyclic loading for further investigation; including the microscopy of cells, immunostaining, and gene expression using multiplex assay method.

### Collagen immunohistochemistry

Cells from the experimental groups and corresponding control group were fixed on the STREX chamber stretchable substrate in methanol for 15 min at room temperature. Hydrogen peroxidase was then used to block endogenous peroxidase activity and reduce non-specific background. Primary antibodies i.e. rabbit anti-collagen type I or rat anti-collagen type III (Merck, Germany) diluted at 1:100 was applied to each specimen and incubated for 1 h. This was followed by the incubation with streptavidin-peroxidase secondary antibody (Dako, Denmark) for 30 min. Finally, the samples were developed with 3,3’-diaminobenzidine tetrahydrochloride chromogen substrate for 30 min and examined under light microscopy (Nikon Eclipse TE2000-S; Nikon Corporation, Tokyo, Japan). All staining procedures were performed in a humidified chamber at room temperature.

### N-cadherin and fibronectin immunofluorescence

The cells were fixed with 3.7% paraformaldehyde followed by a permeabilization process using cold acetone. The cells were then blocked with 1% bovine serum albumin for 30 min, and incubated with N-cadherin or fibronectin antibody (Abcam, Cambridge, UK) at 1:300 dilutions in phosphate buffered saline (PBS) for 1 h at room temperature. The cells were then washed in PBS, incubated with fluorescein isothiocyanate (FITC) secondary antibodies (Abcam, Cambridge, UK) at a concentration of 1:600 dilution for 1 h in the dark, and counterstained for nuclei with Hoechst (Molecular Probes, Oregon, USA) for 10 min. Stained cells were analysed under a laser scanning confocal microscope (Leica TCL SL, Germany).

### RNA extraction and multiplex gene expression assay

To determine the correlation between the tenogenic differentiation potential of hMSCs as the result of mechanical stimulation and Ca^2+^ blocking activity, we used multiplex gene expression assay. The cells were lysed using lysis buffer (Qiagen, Canada) with 1% β mercaptoethanol. Total ribonucleic acid (RNA) was extracted per the manufacturers’ instructions using the RNeasy mini kit (Qiagen, Canada) and stored at -80°C until further processing. Quantity and quality of RNA were analyzed using a spectrophotometer (Nano-Photometer, Implen, München, Germany), and a BioAnalyzer (Model 2100, Agilent Technologies, USA). Only samples with high quality were selected for microsphere-based multiplex branched DNA downstream analysis. The quantitation of mRNA expression was carried out according to the manufacturer’s instructions using the QuantiGene 2.0 Plex assay (2.0 plex set 12082, Panomics/Affymetrix Inc., Fremont, CA, USA). Individual bead-based oligonucleotide probe sets specific for each gene (Table 1) examined were developed by a selected licensed manufacturer (Panomics/Affymetrix Inc., Fremont, CA, USA). In determining the optimal housekeeping gene, we compared *PGK1* (phosphoglycerate kinase 1), *HPRT1* (hypoxanthine phosphoribosyltransferase 1), and *TBP* (TATA box binding protein), and observed that *PGK1* was the most stable housekeeping gene in our experiments [19].

**Table 1:** The tenogenic differentiation genes of interest were determined in this study.

Related marker	Gene name	Abbreviation
ECM component	Collagen type I, α1	*COL1*
	Collagen type III, α1	*COL3*
	Decorin	*DCN*
Tendon lineage	Tenascin C	*TNC*
	Scleraxis homolog A	*SCX*
	Tenomodulin	*TNMD*
Housekeeping gene	Phosphoglycerate kinase 1	*PGK1*

In order to determine whether the inhibition of Ca^2+^ by gadolinium may affect apoptosis of the cells during differentiation during mechanical stretching, a similar method as that mentioned previously was carried out by using the QuantiGene 2.0 Plex assay (2.0 plex set 12314, Panomics/Affymetrix Inc., Fremont, CA, USA) (Table 2). Values for mRNA transcript levels were normalized to corresponding *GAPDH* (Glyceraldehyde 3-phosphate dehydrogenase) values, as was compared to *TBP* and *ACTB* (actin beta).

**Table 2:** The apoptosis genes of interest were determined in this study.

Related marker	Gene name	Abbreviation
Extrinsic pathway	Caspase 3, apoptosis-related cysteine protease	*CASP3*
	Caspase 9, apoptosis-related cysteine protease	*CASP9*
	Fas (TNF receptor superfamily, member 6)	*FAS*
	Tumor necrosis factor (ligand) superfamily, member 6	*FASLG*
Intrinsic pathway (pre-apoptotic)	BCL2-associated X protein	*BAX*
Intrinsic pathway (anti-apoptotic)	B-cell CLL/lymphoma 2	*BCL2*
Others	Calpain 1, (mu/l) large subunit	*CAPN1*
	Calpain 6	*CAPN6*
	Inositol 1,4,5-triphosphate receptor, type 1	*ITPR1*
	Catalase	*CAT*
	Cytochrome b-245, beta polypeptide (chronic granulomatous disease)	*CYBB*
	NADPH oxidase 4	*NOX4*
	Cytochrome c-1	*CYC1*
	Nuclear factor of kappa light polypeptide gene enhancer in B-cells 1 (p105)	*NFKB1*
	Apoptosis-inducing factor, mitochondrion-associated, 1	*AIFM1*
	Poly (ADP-ribose) polymerase family, member 1	*PARP1*
	Poly (ADP-ribose) polymerase family, member 2	*PARP2*
	Poly (ADP-ribose) polymerase family, member 4	*PARP4*
	Mitogen-activated protein kinase 1	*MAPK1*
	Superoxide dismutase 1, soluble (amyotrophic lateral sclerosis 1 (adult)	*SOD1*
	Nuclear factor of activated T-cells, cytoplasmic, calcineurin-dependent 1	*NFATC1*
	Chromosome 6 open reading frame 4	*TRAF3IP2*
	TNF receptor-associated factor 6	*TRAF6*
	Glutathione peroxidase 1	*GPX1*
	Protein tyrosine kinase 2	*PTK2*
	Calcium/calmodulin-dependent serine protein kinase (MAGUK family)	*CASK*
Housekeeping gene	Glyceraldehyde 3-phosphate dehydrogenase	*GAPDH*

### Statistical analysis

Data were presented as mean ± standard deviation or mean error. For nuclei count experiment, Student’s t-test (two-sided, unpaired) was carried out to compare the differences in mean values. While the other experiments, statistical significance was analysed by one-way analysis of variance (ANOVA). A *p* value of < 0.05 was considered statistically significant for all statistical tests.

## Results

### Identification of hMSCs

The shape of the cells from P0 to P3 was shown morphologic characteristics of human bone marrow stromal cells, including fibroblastic-like, spindle-shaped, and plastic adherent (data not shown). The flow cytometry analysis showed that hMSCs exhibited positive staining for CD29 (96.5%), CD44 (97.1%), CD73 (96.6%), CD90 (99.2%), and CD105 (97.7%), while negative staining for CD14 (2.8%), CD34 (0.2%), CD45 (1.3%) and HLA-DR (2.7%) confirmed the phenotype of hMSCs. The cells showed the ability for tri-lineage differentiation (data not shown). Our results demonstrated that hMSCs could successfully commit towards osteoblast lineage visualized by Alizarin Red S staining (for calcified matrix); adipocyte lineage observed by Oil Red O (for lipid droplets); and chondrogenic lineage in the pellet culture system demonstrated positively by Safranin O staining (for cartilaginous matrix). Hence, based on the results of cell identification analyses, it became assured that the isolated stem cells were MSCs.

### Appropriate gadolinium concentration as SACC inhibitor

**Fig 1:**
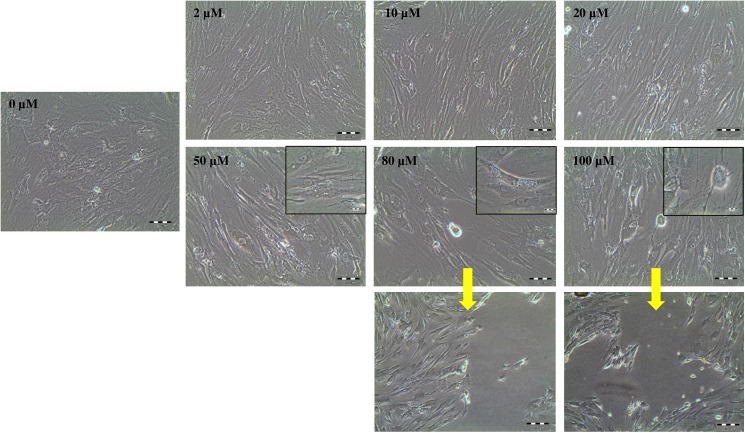
Effects of different gadolinium concentration on hMSCs. Morphological changes of hMSCs cell culture after 72 hours incubation of gadolinium. By increasing the gadolinium concentration, small vesicles were observed (probably apoptotic bodies) as well as cell detachment (the yellow arrow).

**Fig 2:**
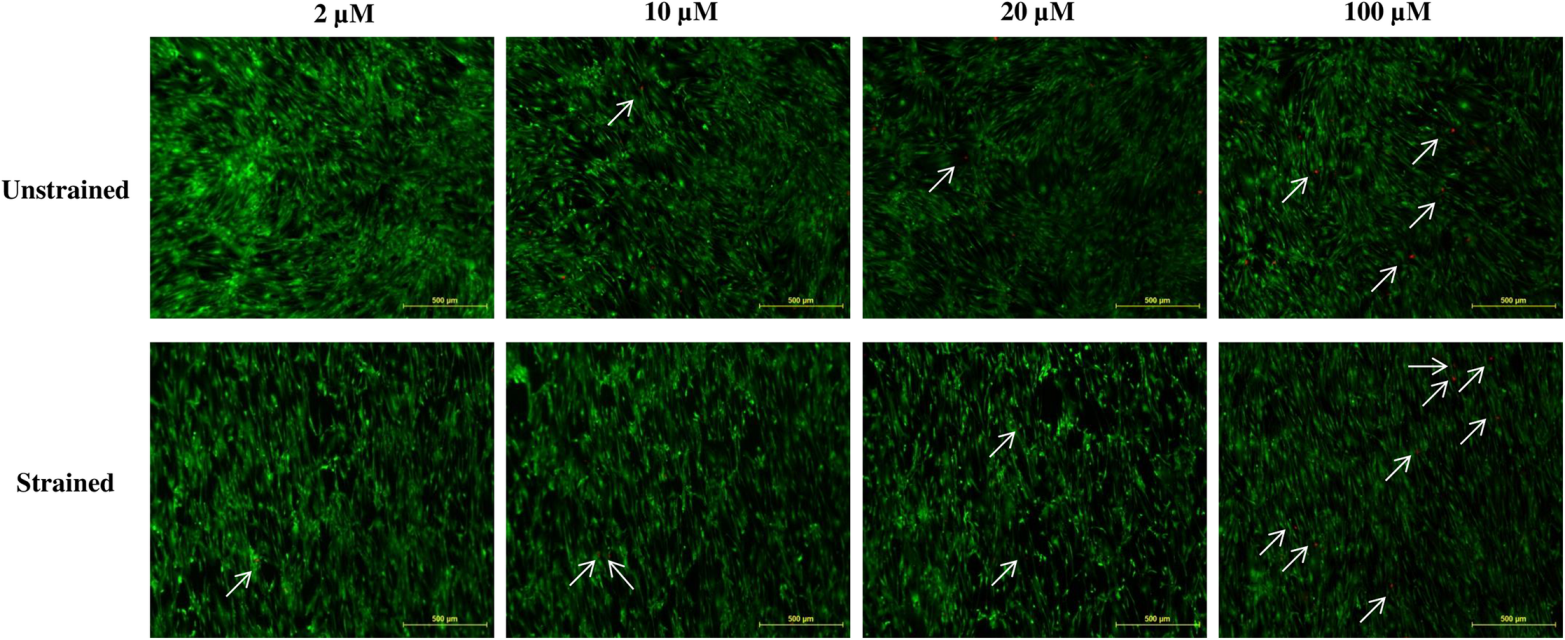
Live (green) and dead (red) cells on hMSC treated with different concentration of gadolinium. White small arrows indicate dead cells. The number of dead cells increased by increasing the SACC blocker concentration.

Unstrained hMSCs were treated with different concentration of gadolinium (2, 10, 20, 50, 80 and 100 μM) to identify the optimal concentration of gadolinium without inducing morphological changes or cell detachment in the silicon chamber (Fig 1). Cells treated at the concentration of 2 μM and 20 μM showed normal appearance of MSCs with similar cell numbers to that of cells in untreated wells. Cells treated with a concentration above 20 μM demonstrated changes from their fibroblastic morphology and reduced cell number, obviously at higher concentration of 80 μM and 100 μM, where cell death and cell detachment became apparent. Similar for the result of live/dead cells experiment, the number of dead cells (red colour) appeared to increase by increasing the SACC blocker concentration (Fig 2). Based on these results, 20 μM concentration of gadolinium was then used for the following experiments.

### Cell morphology following SACC inhibition and mechanical stimulation

**Fig 3:**
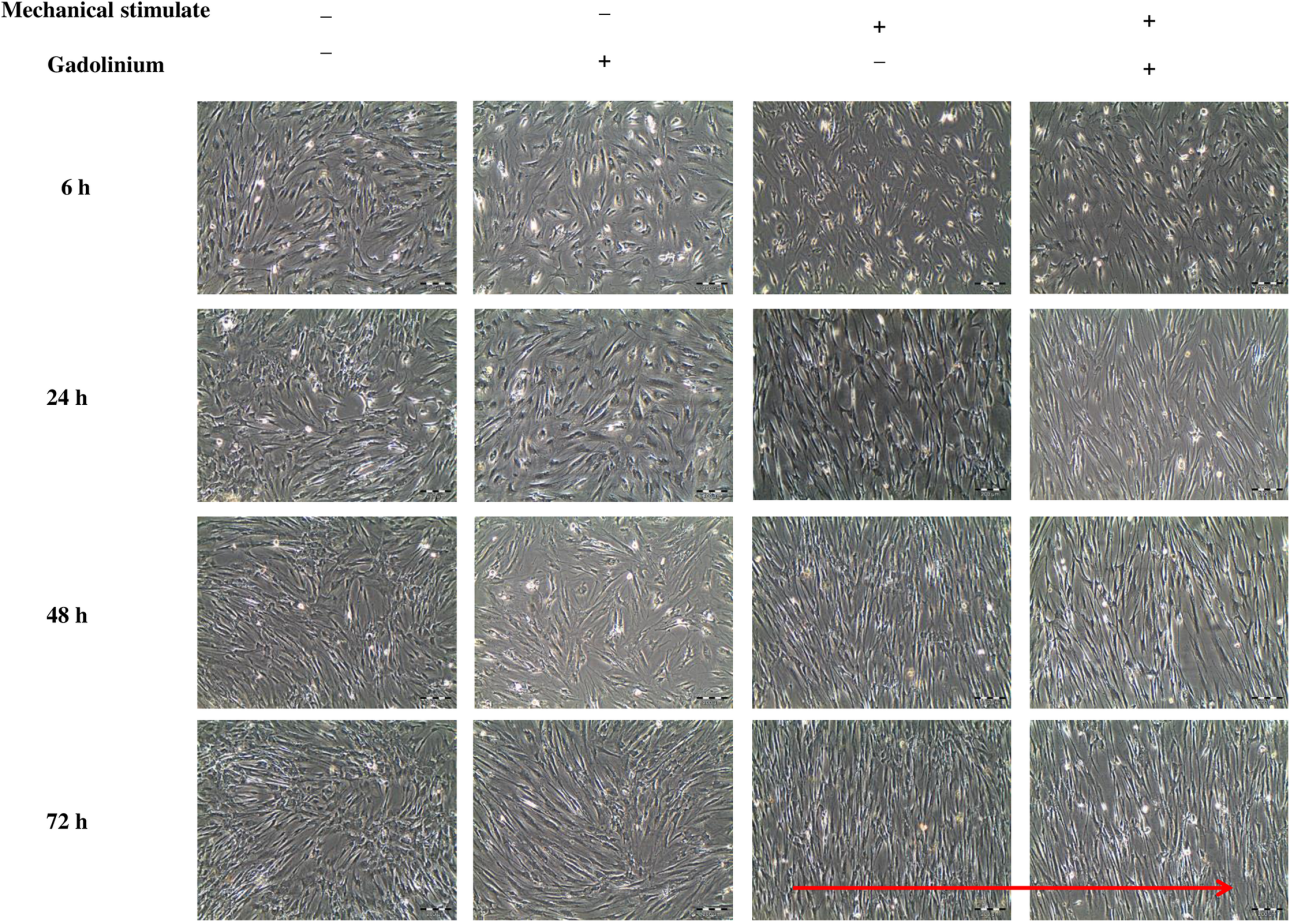
Morphology of hMSCs after treated with gadolinium. The unstrained cells and strained cells at 1 Hz, 8%, at different duration of stretching exposure, with or without using 20 μM gadolinium, respectively. The direction of uniaxial strain is indicated as red arrow.

The morphology of SACC inhibited hMSCs showed no significant difference with non-SACC inhibited hMSCs at the same time points (Fig 3). However, the strained cells treated with SACC blocker showed some changes in the cell numbers.

### Changes in ECM production during stretching and blocking of SACC

**Fig 4:**
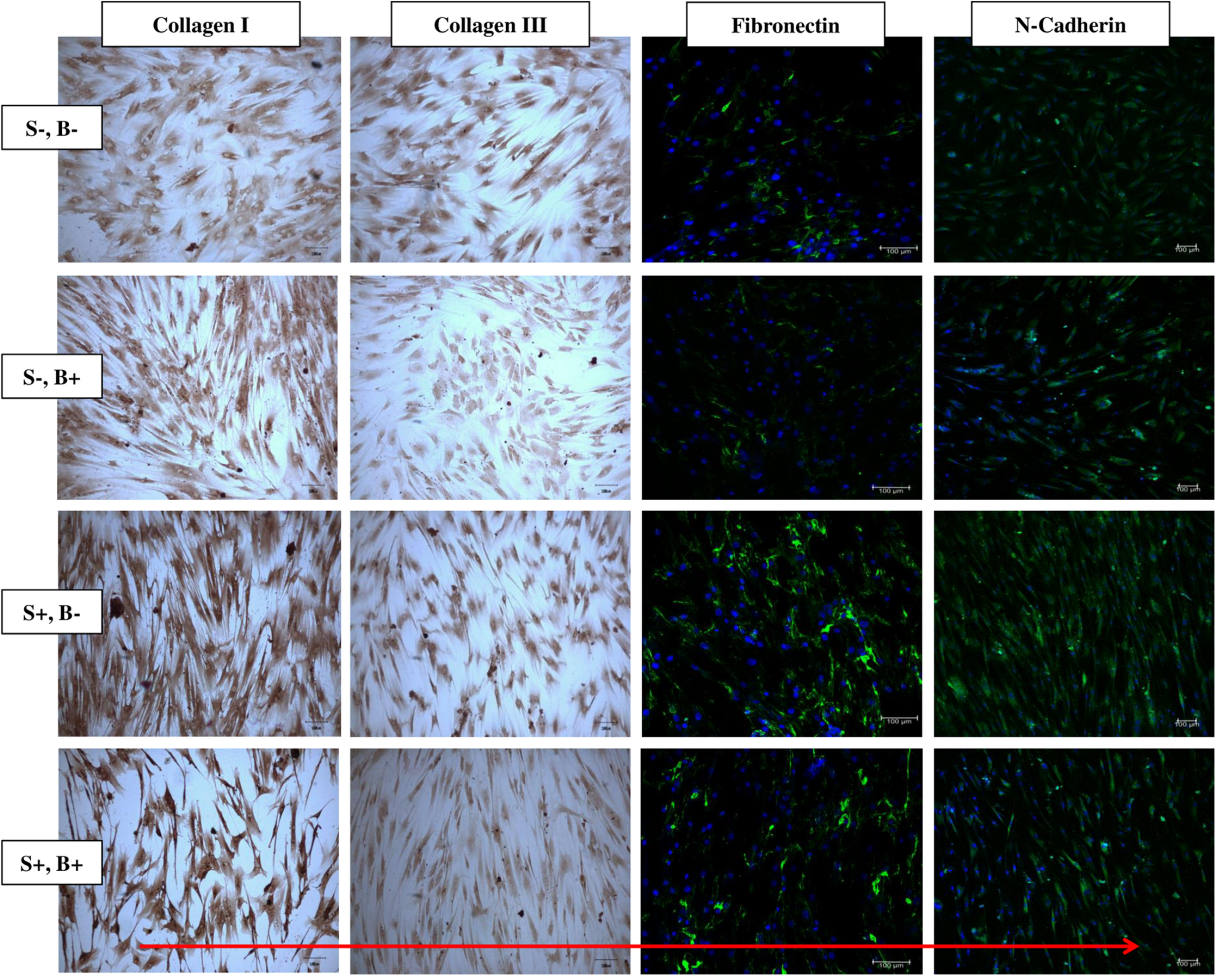
Immunostaining and immunofluorescence images of unstained and strained hMSCs cultured with or without gadolinium. The cells were stained with immunostaining antibody collagen I and collagen III. Immunofluorescence antibody was used as a method to access fibronectin and N-cadherin. Cells were stained with Hoechst (blue) to reveal the nucleus, and the images were merged with the corresponding fibronectin or N-cadherin (green). The direction of uniaxial strain is indicated as red arrow. S-: no mechanical stimulation; S+: cyclic stretching applied; B-: no gadolinium; B+: with SACC inhibitor, gadolinium.

Fig 4 shows the immunostaining of collagen I, collagen III, fibronectin and N-cadherin on both unstrained and strained cells treated with or without gadolinium. The expression of collagen III was found to be decreased in both unstrained and strained cells treated with gadolinium compared to cells without gadolinium treatment. Without SACC blocker, the expression of fibronectin and N-cadherin was increased in strained cells as compared to unstrained cells. However, the intensity of FITC positive cells were relatively less between the strained and unstrained groups when compared to the gadolinium treated cells. Within the group of strained cells, using gadolinium resulted in the reduction in the production of ECM fibronectin and N-cadherin. These results suggest that the ECM production correlates with the presence of SACC in hMSCs. It also suggests that the inhibition of SACC may have resulted in inadequate cell-matrix interaction due the disruption of ECM formation.

### Influence of SACC inhibition on tenogenic differentiation

**Fig 5:**
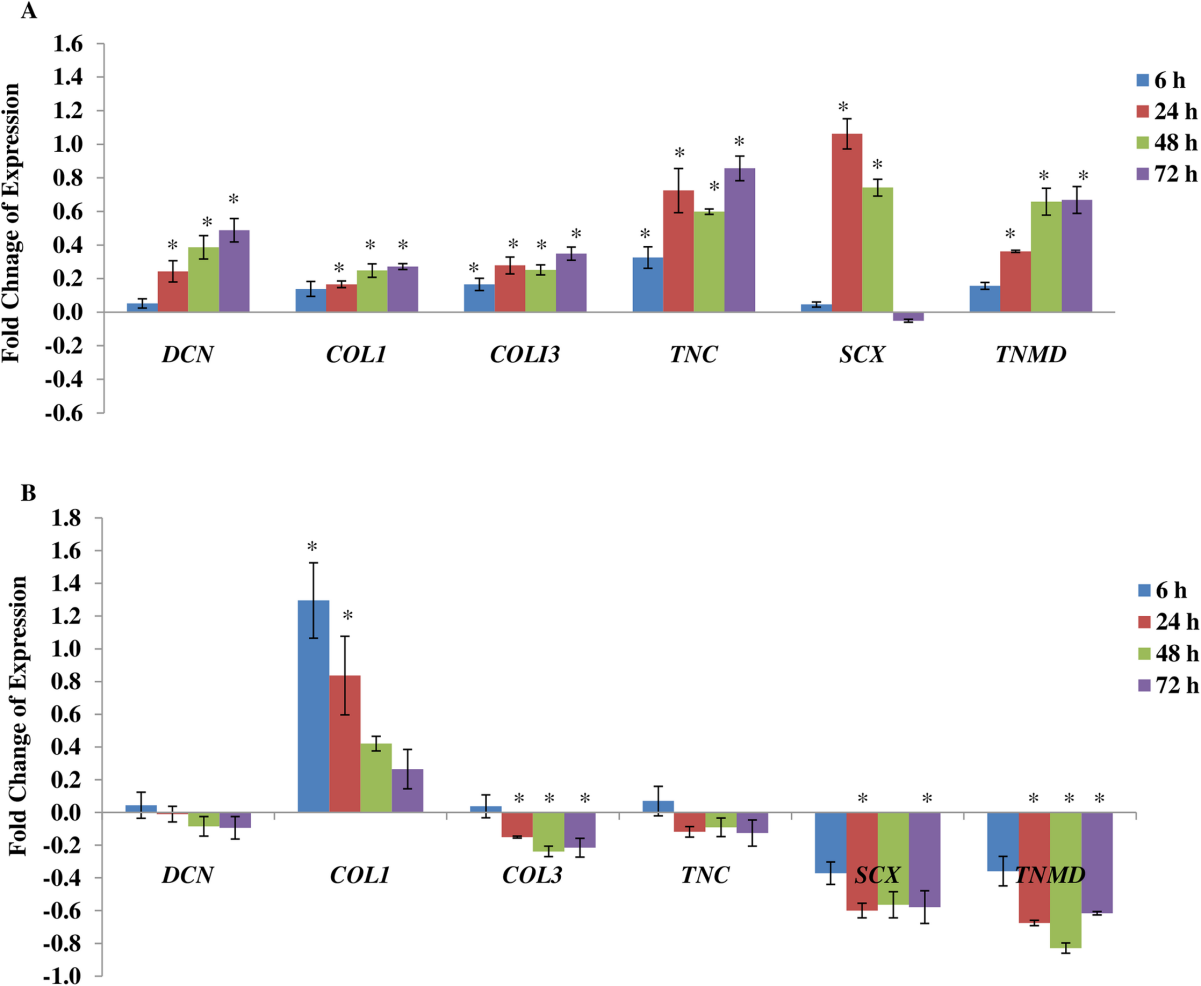
Effect of uniaxial stretching on mRNA expression of tenogenic markers in hMSCs, which were performed at 1 Hz + 8% in different duration of mechanical stimulate. (A) Tenogenic differentiation of hMSCs is triggered by mechanical stimulation. Fold changes of expression were measured by normalizing the relative expressions with the corresponding control groups (unstrained groups). Statistical significance (*p* < 0.05) was represented by asterisk which compared to unstrained. (B) Tenogenic lineage genes expression was influenced after adding SACC blocker, Gd^3+^ to the strained cells. The value of fold change was presented as ratio of strained group treated with gadolinium to strained group without gadolinium. Statistical significance (*p* < 0.05) was represented by asterisk which was compared to strained group without treatment (indicated as 0). N = 6, n = 3. Error bar = ± 1 SD.

Our previous study demosntrated that mechanical stimulation can trigger the tenogenic differentiation of hMSCs [19] (Fig 5A). We then evaluated as to whether inhibiting SACC can influence the ability of mechanical stimulation induced differentiation of hMSCs. Although the cells were mechanically stimulated, blocking SACC has resulted in the decreased expression of tenogenic markers (Fig 5B). *DCN* and *COL3* were down-regulated after 6 h, most significantly for *COL3*. Interestingly, inhibiting SACC does not result in the down-regulation of *COL1*, although the expression levels appeared lower over time. *DCN* and *TNC* were slightly increased at 6 h, as compared to strained cells without SACC blocker. This increase nevertheless declined gradually at later time points. The other two specific tenogenic genes markers, *SCX* and *TNMD*, down-regulated significantly after 24 h. This suggests that SACC might play important role in the differentiation pathway, especially tenogenic lineage.

### SACC inhibition did not induce hMSCs apoptosis

**Fig 6:**
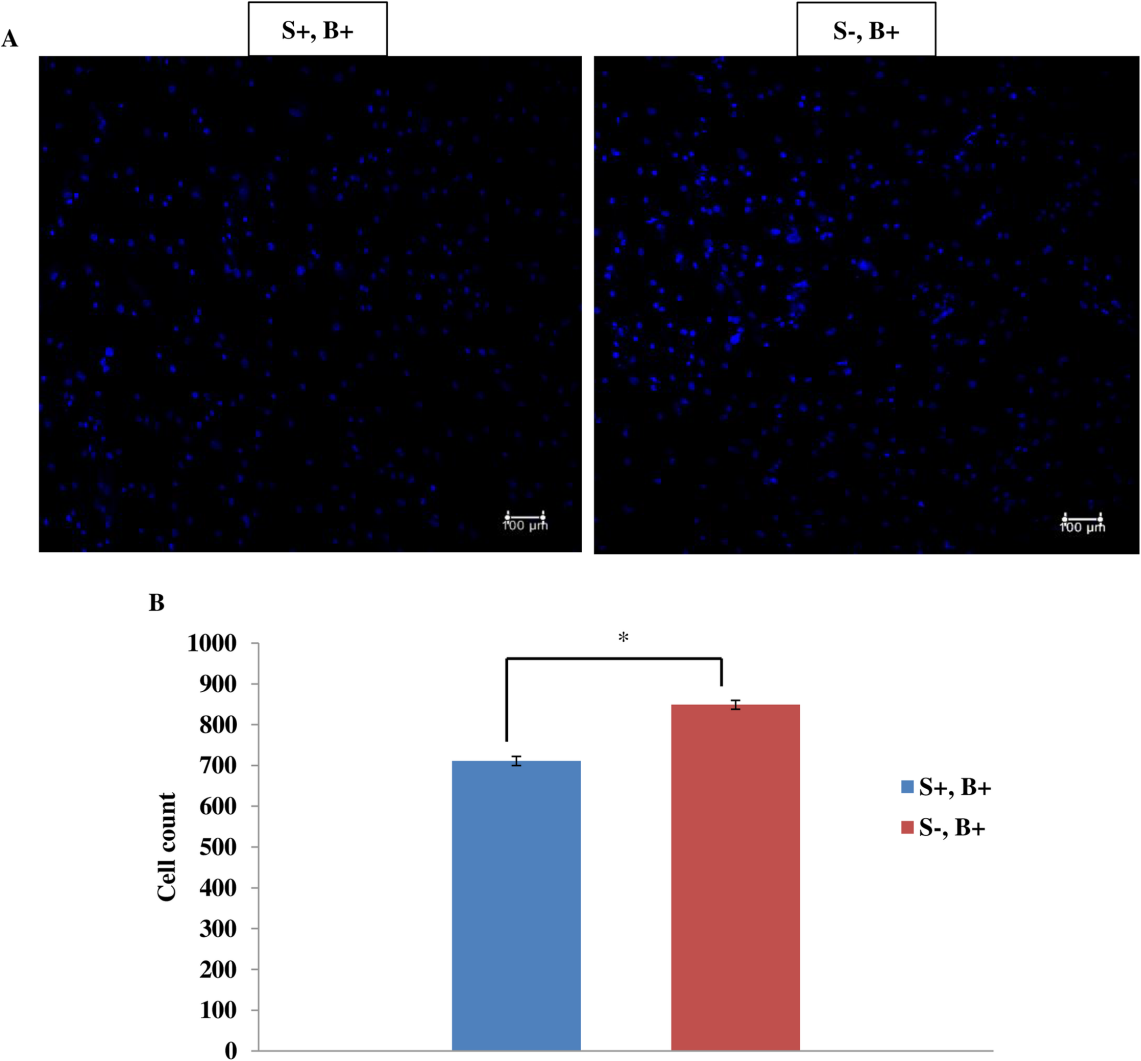
Effect of gadolinium on strain-induced decrease in DNA counts. The cells were fixed and DNA stained by Hoechst (blue). (A) The nuclei of the cells were visualized using confocal microscope and counted. (B) In the presence of Gd^3+^, 8% + 1 Hz for 72 h significantly decreased DNA count. The result was expressed as a mean ± 1 SD for five randomly selected fields in 3 independent experiments. Statistical significance (*p* < 0.05) was represented by asterisk which was compared to unstrained group with gadolinium treated. S-: no mechanical stimulation; S+: cyclic stretching applied; B+: with SACC inhibitor, gadolinium.

**Fig 7:**
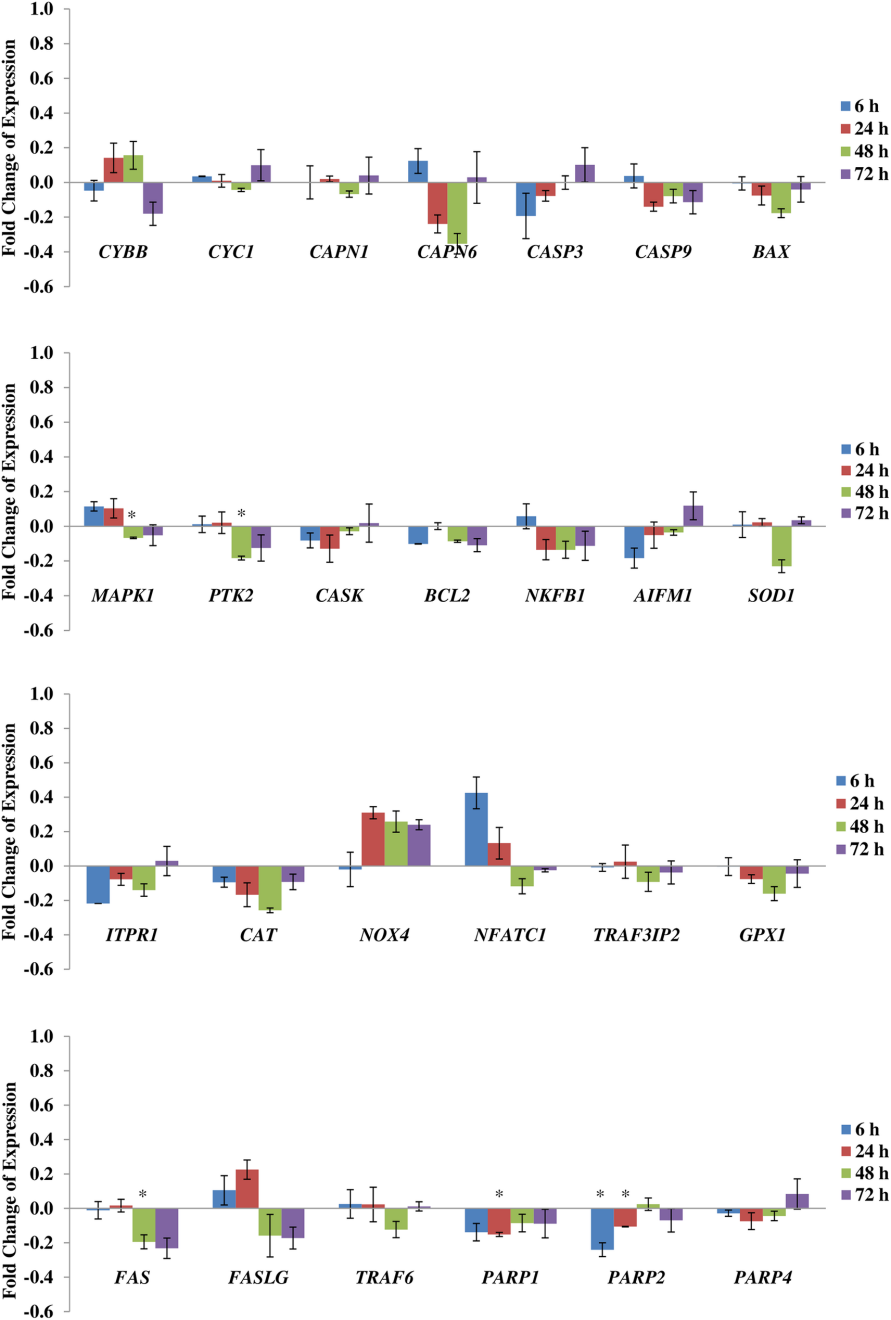
mRNA expression of apoptosis genes subjected to cyclic tensile loading at 1 Hz and 8% at different duration of stretching. The expression level of each gene was normalized with the level of housekeeping gene. The value of fold change was presented as ratio of strained group to unstrained group, where both groups treated with gadolinium. Statistical significance (*p* < 0.05) was represented by asterisk which compared to unstrained group (indicated as 0). N = 6, n = 3. Error bar = ± 1 SEM.

When cells were stained with Hoechst, it showed significant reduction (approximately 16%) of cell number in the strained cells that is treated with SACC blocker (Fig 6). Based on the results of Hoechst staining, we anticipated whether the cell death could be due to apoptosis here, we studied the possibility of the SACC inhibited cells subjected to stretching was driven to apoptotic. Fig 7 shows that by blocking SACC is not inducing apoptosis of hMSCs.

## Discussion

The present study used an *in vitro* mechanical stimulation model system in which isolated hMSCs are cultured on type-I collagen coated silicone membrane to determine if tenogenic differentiation are influenced by SACC activity, which indirectly indicates that calcium signalling may be involved. Some studies have employed this model to investigate hMSCs mechanotransduction in influencing cell deformation in certain controlled microenvironment changes, such as pH and osmotic changes [23–25]. Our study demonstrates that cyclic tensile loading on hMCSs produces an up-regulation of collagen synthesis. In addition, this also results in the transformation of stromal cells into tenogenic-like cells, which is dependent on both the applied frequency and amount of strain subjected to these cells [19]. However, despite these observations, the essential fundamental understanding underpinning the regulation of the mechanotransduction pathways remain unclear. In our attempt to prove our hypothesis that stretch-activated channel may be involved in this process, we investigated the effect of the cyclic uniaxial mechanical stimulation on SACC by using gadolinium ion to block the channel, i.e. because gadolinium is a commonly used antagonist of SACC [26]. The calcium influx by the mechanical potential appeared to be sensitive to the presence of the gadolinium ion. As predicted, Gd^3+^ being a SACC blocker, reduces the differentiation responses of hMSCs. Our study shows for the first time that Gd^3+^ counteracts the tenogenic differentiation effect of hMSCs as the result of uniaxial cyclic mechanical stretching. Nevertheless, it was interesting to note that cell morphology and expression of genes related to apoptosis was not altered significantly. The results of our study confirms that applied uniaxial cyclic load (1 Hz + 8%) with channel blocker does not induce cell death but instead delayed the differentiation process which indirectly indicates that SACC plays a role in the differentiation process of hMSC into tenogenic like cells.

Mechanotransduction, the transmission and conversion of a mechanical stimulus into a biological response, through a variety of mechanisms of which in the present study suggests that it is channel dependent. There are three main stages of mechanotransduction: mechanocoupling, cell-to-cell communication, and effector cell response. Mechanocoupling is the process by which the applied load is transmitted through the tissues and cells, resulting in different types of cellular deformation. Stretch-activated calcium channels have been shown to be involved quite extensively in the mechanotransduction process, demonstrating varied responses as different types of mechanical loading are exerted to cells and tissues. This has been reported in many mesenchymal derived cells such as chondrocytes and MSCs [27–29]. Despite cells of similar lineage i.e. mesenchymal in origin, different cells depend on different types of calcium channel, mainly because of the different functions of these cells. For example, unlike osteoblasts, Ca^2+^ secretion by MSCs does not occur via L-type Ca^2+^ channels but rather, it is mediated by inositol 1,4,5-trisphosphate receptors (InsP_3_Rs). Further, its entry is controlled by store-operated Ca^2+^ channels and not through the efflux of calcium [30]. In general, ion movement in and out of cells response to applied mechanical loading in an almost similar manner despite the differences in their destined functions. It is usually the case that with mechanical stimulation such as stretching, SACs open to allow ions like Ca^2+^, Na^+^, K^+^, and Mg^2+^ to pass through, transducing mechanical signals towards the activation of intracellular signalling molecules [13, 31–32]. Furthermore, integrins, the cytoskeleton, and Ca^2+^ channels have previously been proposed to interact with one another [13, 33]. It is shown that integrin binding and cytoskeletal organization regulate the mechanotransduction process of stretching, which suggests a possible role for Ca^2+^ signalling in this process. However, whilst these signals may regulate the process of cell proliferation and differentiation through specific pathways such as focal-adhesion kinases (FAK) or mitogen-activation phosphorylation kinases (MAPK) pathways, in aberrant or extreme mechanical loading conditions, the increase in intracellular Ca^2+^ can trigger the activation of the calpain family of regulatory proteases that help to drive signalling transduction pathways forward to initiate a downstream pro-apoptotic signalling process [34].

Changes in cell morphology are an important feature to be observed, since the deformation of cell shape is implicated in the translation of mechanical forces into a biochemical response. This may result in the opening of ion channels such as SACC, and the activation of transmembrane signalling proteins such as integrins and G-protein coupled receptors [13]. The mechanically stimulated cell may also spread the signal to adjacent cells (cell-to-cell communication, involving the passage of calcium ions via gap junction, for instance) thereby amplifying the response [35]. In response to elevated intracellular calcium and other signals, enzyme activity is initiated within the cell (e.g. activation of MAPK family members) leads to gene transcription and the production of protein which can include newly synthesized extracellular matrix, or autocrine/paracrine substances like TGFβ or IGF-I which further amplify the adaptive responses [36]. This then suggests that ionic changes, in this case calcium being a widely accepted second messenger, may be heavily involved in the process. It is interesting to note however that Liu et al [37] reported that greater extracellular Ca^2+^ concentrations did not change cell proliferation but significantly inhibited MSCs differentiation.

A study of Coirault et al. [38] demonstrated that effects of Gd^3+^ were not due to inhibition of voltage-gated Ca^2+^ channels. A selective stretch-activated channel blocker would allow the relative contribution of stretch- and voltage-activated channels to tension development to be quantified more precisely. At present, there have been no such blocker available that has a similar function to gadolinium, and as such, gadolinium is presently the only prescribed blocker of stretch-activated channels [39–41]. In previous studies, cation channels are blocked using Gd^3+^, in concentrations of 10–100 μM, depends on cell types [38, 42–45]. Incubation of cells with high concentrations of Gd^3+^ blocks electrical stimulation-induced concentration of intracellular calcium increases, as well as inhibiting the influx and efflux of calcium [40]. The study further reveals that electrical stimulation-induced mechanical deformation of cells may also be prevented by blocking SACCs and other Gd^3+^-sensitive pathways. In terms of proliferation and differentiation, a previous study noted that the inhibition of ERK1/2 phosphorylation by mechanical stretching was dependent on the Gd^3+^ concentrations [46]. A moderate dose of Gd^3+^ (20 μM) was chosen in our study to treat hMSCs to avoid non-specific (or toxic) effects where possible. It is important to recognize that gadolinium ions have a high affinity to bind free bicarbonate and phosphate ions present in several physiological solutions [47]. However, in culture medium (including DMEM used in this study), the phosphate and bicarbonate ions exist as protonated anions that have very low affinity for gadolinium ions. Nevertheless, the possibility that some gadolinium ions are neutralized in the culture medium due to their binding to phosphate and bicarbonate anions cannot be ruled out [41]. Based on our previous literature, the SACC inhibition was assumed to be occurred with 20 μM of gadolinium. A study of Pingguan-Murphy et al [44] showed that isolated articular chondrocytes in unstimulated agarose constructs exhibited spontaneous characteristic of Ca^2+^ transients, where they explained that this may be associated with several factors including the presence of growth factors in the serum, endogenous release of low levels of ATP, and the generation of oxygen-free radicals caused by laser illumination.

In terms of changes to cell morphology as the result of cyclic loading, the present study corroborates previous findings that cell orientation is altered when subjected to cyclic loading [48–49]. Cell appears to re-orientate in longitudinal axis perpendicular to its original orientation as well as the direction of cyclic loading. This phenomenon appears to be necessary for the reduction of excessive strain applied to the cells. It has been suggested that the mechanisms involved in promoting cellular realignment is dependent on various factors, which includes the rearrangement of intra-cellular stress fibers due to energy dissipation and the fluctuations in the ionic exchange mechanisms such as the depolarization of voltage gated channels [50]. Nevertheless, there is no significant change in cell morphology and orientation was observed when Gd^3+^ was added, indicating that cyclic loading may play an important role in deciding the fate of cell behaviour.

As mentioned previously, one of the earliest effected signalling mechanisms is the fluctuation in intracellular calcium. Rise in intracellular calcium can trigger the activation of the calpain family of regulatory proteases that help to drive signalling transduction pathways forward and have been linked to the initiation of downstream pro-apoptotic signalling. We studied *CAPN1* and *CAPN6*, and the results did not show any significant increase. In fact, *CAPN6* appeared to be down-regulated at 24 h and 48 h. Whilst there may be an apparent increase in gene expression, the increment at 72 h is insignificant, and could be due to cytoskeletal remodelling and signal transduction that occurs due to the mechanical stimulation process [51]. Amongst the many downstream intracellular death-signal transmitting pathways, the MAPK signalling cascade is the most studied and elucidated, and have been shown to control cell differentiation and survival [52]. *MAPK1*, also known as *ERK2* (extracellular signal-regulated kinases 2), appears to be down-regulated in long periods of culture conditions (48 h and 72 h) in our study, indicating that cell differentiation can be regulated by mechanical stimulation. Despite the diversity in pro-apoptotic signalling cascades, most cell death pathways ultimately converge with the activation of caspases [53]. Caspases are specialized proteases that are essential for the physical execution of apoptosis. Apoptosis is usually accompanied by the activation of caspase-3, which is one of the most extensively studied caspases with numerous mechanisms of activation [54], which is involves activation of caspase-9 [55]. Once activated, caspase-3 is directly involved in the sustained induction of DNA damage and the disruption of DNA repair mechanisms. Although *CASP3* increased at 72 h, the increase was non-significant, which suggest that blocking of SACC and with uniaxial stretching does not induced apoptosis mediated cell death. Expression of the pro-apoptotic Bax protein and mitochondrial dysfunction also mediate apoptosis following mechanical stress [56]. This gene expression was down-regulated when mechanical stretching was applied on SACC-inhibited MSCs, demonstrating that mechanical stretching at 1 Hz + 8% did not induce MSCs cell death. Indeed, there is a significant decrease in nuclei count for SACC-inhibited strained cells. However, since there is no apoptosis occurs in this group, we assume that the decrease in nuclei count was due to partial oxidative stress. This was confirmed from the gene expression analysis which revealed a down-regulation of *GPX1* and *SOD1*. This suggests that reduction in these 2 genes may result in excessive reactive oxygen species (ROS) production in SACC-inhibited cells under cyclic stretch. *GPX1* is ubiquitously expressed in many tissues, where it protects cells from oxidative stress. The SOD1 enzyme is an important constituent in apoptotic signalling and oxidative stress [57]. SOD1 inhibits apoptosis by interacting with BCL-2 proteins or the mitochondria itself [58], where was also shown in our gene expression analysis. Study of Tan et al [59] revealed that decreased osteogenesis of adult MSCs by ROS under cyclic stretch by down-regulation of *SOD1*, and proposed manipulation of the cells with antioxidant would improve their osteogenic ability. Blocking SACCs with gadolinium does not appear to protect cells against strain induced apoptosis, indicating that these channels are not involved in the up-regulation of apoptotic pathways in hMSCs. Similar findings were mentioned elsewhere [60].

When reviewing similar studies we note that other than stretching, which was used in the present study, other loading modalities such as tension, compression, fluid flow, and osmotic pressure [28, 44, 61–62], appears to be dependent on Ca^2+^ signalling as well. This indicates that mechanotransduction for tensile loading in hMSCs may be necessary. This was evidenced by the lack of a response to the mechanical load when intracellular calcium was chelated. SACCs play a key role in the mechanoresponse of MSCs to tension, compression, and fluid flow [27–28]; yet, in the current study, their inhibition did not fully suppress the mechanoresponse of the MSCs. These results suggest that the mechanotransductive pathways utilised by MSCs in response to uniaxial tensile loading are distinctive from those used to sense and respond to other loading modalities.

Cells, including MSCs and tenocytes appear to share elements of a load-sensing mechanism similar to osteoblasts and osteocytes; stretch-activated potassium and calcium channels, internal calcium release, interstitial ATP release, and gap junction signalling. These appear to play a role in the proliferative response to membrane deformation, substrate deformation, or fluid shear [63]. A key requirement to triggering the intracellular calcium response in cells under tensile loading is that the calcium must be included in the flow medium, otherwise, the flow by itself does not activate the cells [64–65]. Calcium efflux which occurs locally into cells can potentially alter the osmotic pressure around the hMSCs. Such changes in osmotic pressure might also be responsible for activating the cell by SACC [64, 66–67]. The osmotic effects may come into play by the opening of the SACC to allow the influx of extracellular calcium into the cytoplasm of the cells. The total amplitude of intracellular calcium response elicited by the Ca^2+^ supplemented medium however cannot be explained solely by the increase in the osmotic pressure. In another study, it was demonstrated that gadolinium did not affect the intracellular response in osteoblast [68], which further suggests that SACC is not essential in the mechanisms by which osteoblasts detect the elevation of concentration of calcium. Again, this suggests that differences in cell types may lead to different responses.

Results show that supplementing gadolinium ion into cell cultures reduced the tenogenic differentiation of strained hMSCs. Treatment with this blocker had no effect on unstrained cells, but prevented the increase in signalling associated with cyclic stretching, and eventually influences the cell response. This finding has clear implications on the relationships between calcium with its role in actomyosin contraction of stress fibres, and calcium signalling during tensile loading at 1 Hz + 8% strain. Previous studies in which gadolinium inhibited mechanically induced calcium signalling in mechanically stimulated cells appears to have been observed previously [28, 47, 69]. It is not clear from our literature search as to whether an agonist is available for enhancing the SACC function, which contrasts with the function of gadolinium. If there are, it may not be useful if this can regulate the SACC function positively, thus imitating or even enhance the process of mechanotransduction via chemical methods.

## Conclusions

The results of our study demonstrate that SACC blocker used concurrently with uniaxial stretching partially affects tenogenic differentiation and at prolonged periods, induce cell death. However, the cell death is not apoptosis mediated but could be related to decline in the antioxidant markers. In addition, tenogenic differentiation of bone marrow stromal cells may likely be dependent on calcium stretch-activated channels, especially within the first 24 hours. Using this knowledge, we can therefore assume that future studies looking at manipulating calcium signals via SACC using chemical or mechanical means can be used for enhancing cell differentiation for potential tissue engineering purposes.
